# Bilateral Giant Hydronephrosis in a Ten-Year-Old Male

**DOI:** 10.1155/2018/2470369

**Published:** 2018-04-17

**Authors:** Sowmini P. Kamath, K. Ganesh Pai, B. Shantharam Baliga

**Affiliations:** ^1^Department of Pediatrics, Kasturba Medical College (Constituent Unit of Manipal Academy of Higher Education), Mangaluru 575001, India; ^2^Department of Pediatric Surgery, Kasturba Medical College (Constituent Unit of Manipal Academy of Higher Education), Mangalore 575001, India

## Abstract

We describe a ten-year-old male with bilateral giant hydronephrosis due to pelviureteric junction obstruction presenting with abdominal distension and renal failure. The diagnosis was confirmed on computed tomography and required a two-stage procedure, initially percutaneous nephrostomy followed by Anderson–Hynes pyeloplasty with recovery of kidney function.

## 1. Introduction

Pelviureteric junction obstruction is the usual cause of childhood hydronephrosis. Giant hydronephrosis is defined as either pelvicalyceal system dilatation containing more than one litre of urine [1] or causing hemiabdominal swelling or crossing the midline and covers 5 vertebrae in length [2, 3]. It may present as an asymptomatic abdominal mass. Bilateral giant hydronephrosis in children is rare, and this case highlights importance of reparative surgery to preserve renal functions.

## 2. Case Presentation

**Figure 1:**
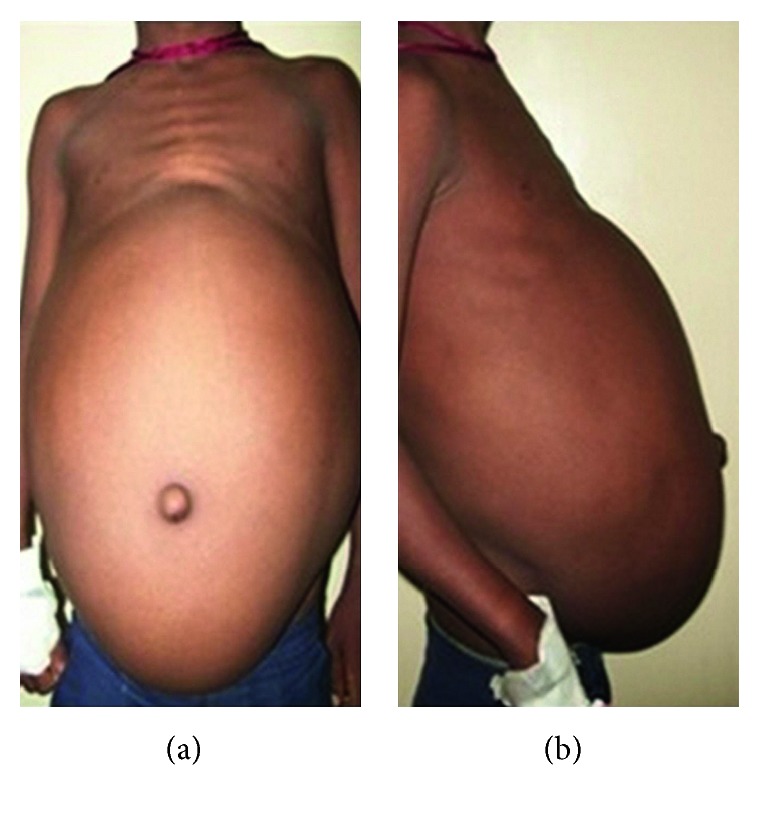
Abdomen grossly distended with a cystic mass.

**Figure 2:**
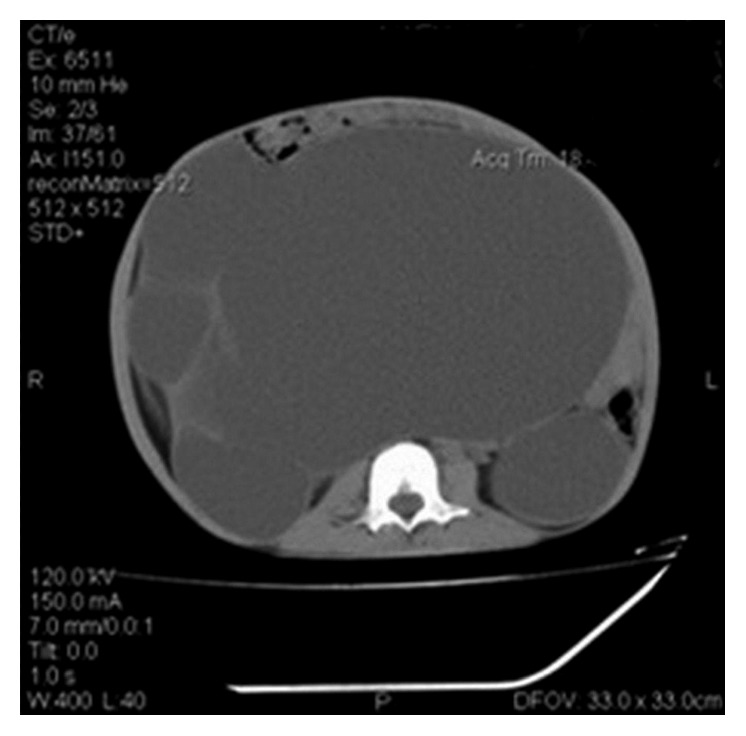
CT abdomen showing the renal mass occupying the entire abdomen.

**Figure 3:**
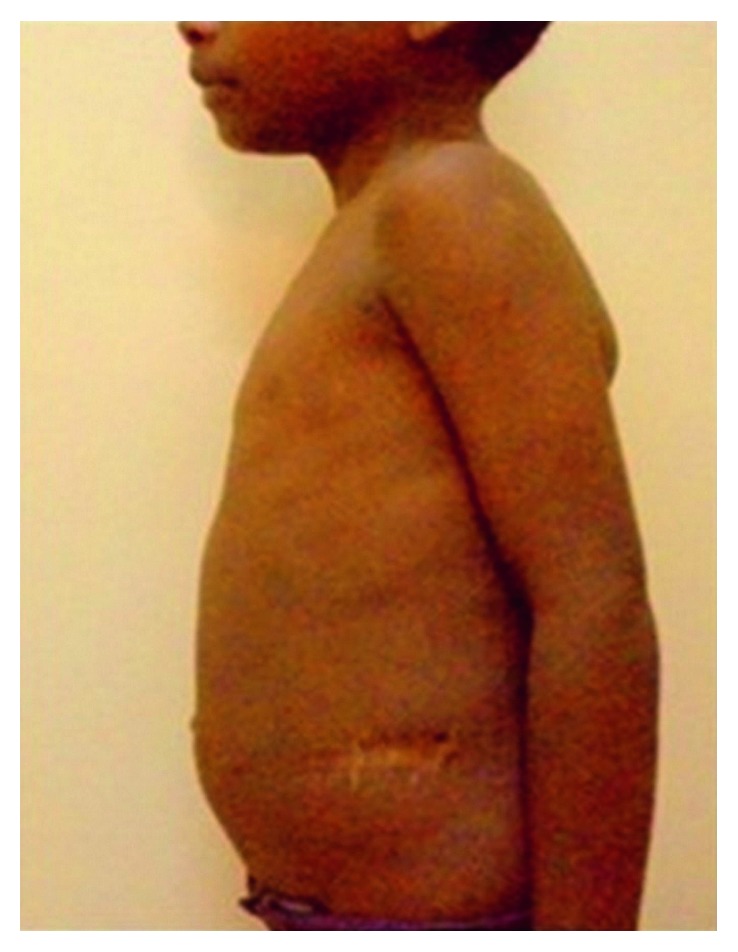
Postoperative picture at recent follow-up.

A ten-year-old male child was admitted with a history of abdominal swelling of 5-year duration. The rest of the history was remarkable for absence of other complaints. His height and weight was between 3rd and 10th centiles as per the CDC chart, and blood pressure was 150/92 mmHg in right upper limb supine position (above 95th centile). On abdominal inspection, he had distension mostly in the lumbar areas, hypogastrium with eversion of umbilicus, flaying of ribs, cystic on palpation, and dull on percussion with absent bruit or rub (Figure 1). At admission, blood urea was 171 mg/dL, serum creatinine 5 mg/dL, serum sodium 140 mEq/L, and potassium 3.4 mEq/L. Urine analysis was normal. Ultrasonogram of the abdomen showed large heterogeneous mass in the lower abdomen with septation and internal echoes. Noncontrast-enhanced computerised tomography (CT) of the abdomen revealed bilateral hydronephrosis with the right side being massively dilated with septation, gross thinning of renal parenchyma and dilatation of the pelvicalyceal system (Figure 2). A percutaneous nephrostomy was placed on the right side, and more than 5 litres of urine was drained. Left nephrostomy was done 2 weeks later followed by improvement in renal functions. After 4 weeks, right Anderson–Hynes pyeloplasty was done. Left pyeloplasty was carried out in another 2 weeks with bilateral double J ureteric stent placement. A revision pyeloplasty was required later using a flap from the renal pelvis in view of failure of the right pyeloplasty. On follow-up, there was no abdominal distension with surgical site being healthy (Figure 3). Also, postoperatively, renal functions improved and remained normal at six months of follow-up with normal blood pressure.

## 3. Discussion

Hydronephrosis can reach surprisingly massive proportions without causing symptoms. Stirling in 1939 defined giant hydronephrosis as presence of more than 1000 mL of fluid in the collecting system [1]. Bilateral giant hydronephrosis in paediatrics is rare. We report a child with bilateral giant hydronephrosis presenting as asymptomatic abdominal mass and having 5 litres of fluid collection.

Crooks et al. [2] did a review of twenty children with giant hydronephrosis, but only one was bilateral. They defined giant hydronephrosis as a kidney that occupied a hemiabdomen, crossed the midline, and was at least 5 vertebrae in length. In majority of patients, the aetiology was pelviureteric junction obstruction [2, 4] as seen in our patient. It is more common in males, usually on the left side, and other causes being urinary stones, congenital anomalies of the urinary tract such as ureteric atresia, or compression of the urinary tract by aberrant vessels or tumours [5].

Giant hydronephrosis occupying the entire abdomen can mimic ascites to a great extent. Paracentesis in these cases could be detrimental causing pyelonephrosis, sepsis, and shock. Other cystic lesions like mesenteric, choledochal cysts (intraperitoneal cysts), renal adrenal, or pancreatic pseudocysts (retroperitoneal cysts) are the differential diagnosis. Each one of these entities could be differentiated by abdominal ultrasonography.

Giant hydronephrosis is usually asymptomatic. Abdominal ultrasonogram is the first diagnostic modality but may be inconclusive, and CT or MRI may be needed [6].

Management of giant hydronephrosis requires a two-stage procedure with initial slow decompression by percutaneous nephrostomy [5]. Harper et al. [7] did a study on laparoscopic nephrectomy for paediatric giant hydronephrosis, but the massive size of the kidney in this case ruled out this approach. In this child, initial nephrostomy was done followed by pyeloplasty. A similar approach was done by Augustin et al. [8] in a 7-year-old with giant hydronephrosis in a single right kidney. This two-stage procedure helps always to preserve residual renal function.

In contrast to our case, nephrectomy of right giant hydronephrosis was done in a 6-year-old male by Sharma et al. [9]. Severe and permanent impairment of renal function may require nephrectomy and renal transplantation. Thus, reparative surgery should always be attempted rather than primary nephrectomy. Renal transplantation procedures may save children with giant bilateral hydronephrosis especially who are beyond the stage of surgical repair.

With advancement in antenatal screening, dilatation of the urinary tract is detected in utero and managed. According to the Consensus statement on management of antenatally detected hydronephrosis [10], surgical correction should be attempted in infants with symptomatic obstructive hydronephrosis, bilateral hydronephrosis, or hydronephrosis in solitary kidney, all with worsening dilatation and deteriorating renal functions. Hence, obstructions of the urinary tract reaching giant proportions may become rare in future.

In conclusion, giant hydronephrosis can present as a painless abdominal swelling. As a close mimic to gross ascites, the condition should be considered as a differential diagnosis with caution prior to procedures such as paracentesis. Early relief of obstruction using a two-stage procedure of nephrostomy followed by pyeloplasty may lead to preservation of renal function.
